# Effects of egg as an early complementary food on growth of 6- to 9-month-old infants: a randomised controlled trial

**DOI:** 10.1017/S1368980023002604

**Published:** 2023-11-29

**Authors:** Hannah Ricci, Mieke Faber, Cristian Ricci, Herculina S Kruger, Linda Malan, Regina Nakiranda, Marina Visser, Cornelius Marius Smuts

**Affiliations:** 1 Centre of Excellence for Nutrition, North-West University, Potchefstroom, South Africa; 2 Africa Unit for Transdisciplinary Health Research (AUTHeR), North-West University, Potchefstroom, South Africa; 3 Non-Communicable Diseases Research Unit, South African Medical Research Council, Tygerberg, South Africa

**Keywords:** Eggs, Infants, Growth and development, Anaemia, Iron status

## Abstract

**Objective:**

To assess the effect of daily egg consumption for six months on linear growth (primary outcome), weight-for-age, weight-for-length, mid-upper arm circumference-for-age, head circumference-for-age Z-scores, gross motor milestones development, anaemia and iron status (secondary outcomes) in a low socioeconomic community.

**Participants:**

Infants aged 6 to 9 months living in the peri-urban Jouberton area, in the Matlosana Municipality, South Africa.

**Design:**

A randomised controlled trial with a parallel design was implemented. Eligible infants were randomly allocated to the intervention (*n* 250) receiving one egg/day and the control group (*n* 250) receiving no intervention. The participants were visited weekly to monitor morbidity and gross motor development, with information on adherence collected for the intervention group. Trained assessors took anthropometric measurements, and a blood sample was collected to assess anaemia and iron status. There was blinding of the anthropometric assessors to the groups during measurements and the statistician during the analysis.

**Results:**

Baseline prevalence of stunting, underweight, wasting, overweight and anaemia was 23·8 %, 9·8 %, 1·2 %, 13·8 % and 29·2 %, respectively, and did not differ between groups. Overall, 230 and 216 participants in the intervention and control groups completed the study, respectively. There was no intervention effect on length-for-age, weight-for-age, weight-for-length Z-scores, gross motor milestone development, anaemia and iron status.

**Conclusions:**

Daily egg intake did not affect linear growth, underweight, wasting, motor milestones development, anaemia and iron status. Other interventions are necessary to understand the effect of animal-source food intake on children’s growth and development. This trial was registered at https://clinicaltrials.gov/ (NCT05168085).

Although the global prevalence of childhood stunting is decreasing gradually, it continues to remain high in Asia and sub-Saharan Africa^(1)^. Being stunted in childhood is associated with an increased risk of morbidity, impaired motor and cognitive development, as well as inability to attain their full developmental potential^(2)^. In addition, it has been previously reported that iron deficiency anaemia in infants and young children prevents them from attaining their full developmental potential, as it increases the risk of stunting and poor motor development outcomes during childhood^(3)^. Stunting has been ascribed to recurrent infections, suboptimal care and poor nutrition of infants and young children^(4)^. Research evidence has shown that the complementary diets of children living in resource-limited settings are deficient in vitamins and minerals, such as iron, zinc, vitamin A and iodine, as well as high-quality proteins necessary for optimal child growth, development and general well-being^(5)^. It was within this context that the international community recommended that animal-source foods be added daily to complementary foods to make up for the protein, vitamins and minerals that are lacking from the usual complementary diets^(6)^.

Chicken eggs (hereafter ‘egg’) have been identified as easily accessible and relatively more affordable in comparison to other animal-source foods^(7,8)^. Eggs are high in nutrients to support growth and development, which may be particularly important for vulnerable children living in resource-limited settings^(8)^. Recent studies have shown that egg consumption during infancy and early childhood can positively influence poor linear growth^(9,10)^. However, there is limited evidence to support the efficacy and effectiveness of egg-related intervention on growth and developmental outcomes of infants and young children living in low- and middle-income countries, including South Africa. There have also been differences in current studies evaluating the effects of egg intake on the nutritional status of infants and young children; some studies reported positive outcomes^(10–13)^, and others did not^(14,15)^. For instance, in the study by Iannotti and co-authors stunting was reduced by 47 % and underweight by 74 % when children aged 6 to 9 months were provided one egg per day for a period of six months^(10)^. However, in the study by Stewart and co-authors there was no effect on stunting and underweight after a six months of egg intervention^(14)^.

Against this background, the primary aim of the present study was to investigate the efficacy of daily egg consumption on length-for-age Z-score (LAZ) and the prevalence of stunting of infants from a low socioeconomic community in South Africa. The secondary aim was to assess the effect of egg consumption on weight-for-age (WAZ), weight-for-length (WLZ), mid-upper arm circumference-for-age (MUACZ) and head circumference (HC)-for-age (HCZ) Z-scores, gross motor milestones development, anaemia and iron status of infants from a low socioeconomic community in South Africa.

## Methods

### Trial design and participants

This study was a 6-month follow-up randomised controlled trial with parallel design conducted in the peri-urban Jouberton area, Klerksdorp, in the Matlosana Municipality in North West province, South Africa. Recruitment and enrolment occurred from 16th February 2021–7th July 2021, while exiting from the study occurred six months from the day of enrolment. The study site has high rate of unemployment, and the typical diet of the infants is maize meal porridge, rice, legumes, sweets and savoury snacks^(16–18)^. Infants were enrolled into the study at the age of 6 to < 9 months if the mother/caregiver resides in the study municipality and if the infant was born a singleton. Infants were excluded if they had severe obvious congenital abnormalities, Hb < 7 g/dl, WLZ < –3, diseases referred for hospitalisation, known allergies or intolerances to egg and receiving special nutritional supplements as part of a feeding programme. Also excluded were mothers/caregivers/legal guardians (hereinafter referred to as mother) planning to move out of the study area within 9 months or if the mother was below 18 years at the start of the study. Recruitment of mother-infant pairs was mainly face-to-face at household level in the local language or in English. Potentially eligible mother-infant pairs received an invite to the central study site for consent, screening and enrolment. The recruitment process continued until the required number of participants was enrolled into the study. Skin prick test was done on all the infants at baseline and endpoint for egg sensitisation and an egg-feeding test at baseline.

### Interventions

The randomised controlled trial consisted of two groups, the intervention group (*n* 250), which was given one egg (grade 1 or about 50 g) per day for six months, and the control group (*n* 250). The duration of the intervention was six months, which is similar to other studies^(10,14)^. Both groups received the same treatment and monitoring, except that the control group did not receive eggs or any food supplement from the study. The study participants in the intervention group received one dozen eggs on a weekly basis (seven eggs for the intervention child and five eggs for household consumption to prevent them from eating the index infant’s weekly ration). All infants whose mothers gave consent and who met the inclusion criteria were exposed to egg at enrolment (skin prick and feeding test) and monitored for egg sensitisation (assessed by using the Childhood Allergy and Immunology Research questionnaire and skin prick test when indicated) during the study.

Trained fieldworkers visited participants weekly to distribute the eggs and to remind the mothers to give one egg per day. Information on adherence to egg intake, morbidity symptoms using a diary, gross motor milestones development using the pictorial chart and a seven-day unquantified food frequency questionnaire, for all the infants, was collected during the weekly visits. All questionnaires had previously been used in a similar study^(19)^ and tested for face validity in the study population. As an incentive, the households of the control group received 5 kg of maize meal monthly for household consumption and 4 dozen of eggs when the infant exited the study. However, the study did not promote the consumption of the maize meal by the study infants. All the eggs were procured from the same farm throughout the study.

#### Sample size calculation

The sample size calculation was done by considering two primary outcomes representing stunting reduction in relation to the intervention. Based on previous studies^(19,20)^, we expected an increase of 0·3–0·6 units and a sd of 1–1·2 units for LAZ. Based on this, we considered an effect size index, *d* = *Z*-_score reduction_/*Z*-_score std_, between 0·3 and 0·6^(21)^ to detect a medium to small difference between the groups under comparison. Another sample size calculation was performed considering a baseline stunting prevalence of 27 %^(18)^ and a target reduction between 30 % and 50 % (relative risk = 0·5–0·7). With the attrition rate set at 25 %, the expectation was a sample size of at least 250 infants per group to have sufficient statistical power to detect an increase in LAZ, effect size index higher than 0·3 units, and to detect a stunting prevalence reduction of 50 % given a baseline prevalence of 27 %.

### Outcomes

Outcome assessment of the trial occurred at three time points, baseline, midpoint (three months from baseline) and endpoint (six months from baseline). In general, all assessments took place at the central study site, except for weekly morbidity, adherence, food frequency questionnaire and follow-up gross motor milestones development assessments. Infants’ date of birth, birth weight and length, and gestational age were recorded from the clinic booklet; in the absence of gestational age in the booklet, maternal recall of gestational age was recorded. Information on household characteristics, including household food insecurity experience^(22)^ and infant feeding practices, was collected at baseline using questionnaires which have been previously used at the study setting. Maternal depression status was assessed using the Edinburg Postnatal Depression Scale (validated for use in South Africa^(23)^). Infants’ dietary intake was also assessed at all three time points using an unquantified food frequency questionnaire (assessed usual food intake in the past seven days using options, such as every day, most days (not every day, 4–6 times/week), once a week (1–3 times/week) and never), which has been used previously at the study setting^(19)^.

#### Anthropometric outcome procedures

Anthropometric data on the infants were collected at all three time points. Infants were undressed and weighed to the nearest 0·01 kg using two standardised digital infant scales (Seca 334 and 727). Recumbent length was measured to the nearest 0·1 cm using an infantometer (Seca 416). Mid-upper arm circumference and head circumference measurements were taken to the nearest 0·1 cm using Seca 201 and 212 measuring tapes, respectively. All measurements were taken in duplicate. If the values of the duplicate measurements differed by more than 0·01 kg for weight, 0·5 cm for length, or 0·2 cm for mid-upper arm circumference and head circumference, a third and/or a fourth measurement was taken, and the two closest values were recorded. In the case where a baby could not lie or sit still, the indirect method was used, where mothers were weighed with and without the infant. The mothers’ height and weight were taken at baseline to assess their BMI. The anthropometric measurements of the infants were converted to LAZ, WAZ, WLZ, MUACZ and HCZ using WHO child growth standard specific for age and sex^(24)^. Stunting was defined as LAZ < –2, underweight as WAZ < –2, wasting as WLZ < –2 and overweight as WLZ > +2^(17)^.

#### Gross motor milestone development outcome procedures

Information on developmental milestones was collected at baseline and during the weekly home visits, using a 14-item (pull to sit, creep 1, sit 1, sit 2, all fours, creep 2, crawl, stand 1, walk 1, stand 2, walk 2, run, jump and stand on one foot) pictorial chart based on the WHO’s standards^(25)^. The date on which a mother observed a particular milestone was recorded on the pictorial chart. This information was used to determine the age and duration for the infants to attain a specific milestone. The WHO’s windows of achievement for the 10th (stand 2 – able to take a few staggering walking steps without support) and 11th (walk 2 – able to walk properly without support) milestones were taken as the overall event for analysis as they provide a good number of events^(19)^.

#### Anaemia and iron status outcome procedures

A trained professional nurse collected capillary blood samples by means of finger and/or heel prick at baseline and endpoint. Hb was measured on the day of blood collection using a portable Hb HemoCue Hb 201+ system (Angelholm, Sweden). In addition to these, blood samples were collected into lithium heparin Microvette® CB 300 (Sarstedt), centrifuged for 10 min at 2000 g and separated for plasma, aliquoted into 0·2 ml Eppendorf tubes, cooled to ∼6°C and transported on the same day on ice packs in a cooler for storage at –80°C at the Centre of Excellence for Nutrition’s laboratory until analysis. Plasma ferritin (PF) to assess body iron stores and plasma soluble transferrin receptor (sTfR) and inflammatory markers, C-reactive protein and *α*-1-acid glycoprotein were measured using the Quansys Bioscience Q-Plex™ Human Environmental Enteric Dysfunction (11-Plex) multiplex sandwich assay technique^(19,26)^. Anaemia was defined as a Hb < 11 g/dl, iron deficiency (ID) as PF < 12 μg/l, iron deficiency anaemia (IDA) as both PF < 12 μg/l and Hb < 11 g/dl and iron deficiency erythropoiesis (IDE) as plasma sTfR > 8·3 mg/l^(27–29)^. The presence of inflammation was defined as C-reactive protein > 5 mg/l and *α*-1-acid glycoprotein > 1 g/l^(30)^.

#### Randomisation

There was a random assignment of eligible infants in a 1:1 ratio. A randomisation sequence of pseudo-random numbers, generated by the RANNOR function of the SAS software package version 9·4, generated the allocation codes. There was a dataset with a list of 500 tags (250 tags for each group) generated. This list of tags merged into a sequence of random numbers generated from an underlying normal distributed variable. The sorting of the generated random numbers was so that there was a random list of codes and tags obtained; this was used to randomly assign an equal number of 250 participants per group.

#### Blinding

Due to the nature of the study, the intervention was not blind to field staff, the nurse and mothers; however, there was blinding of all questionnaire and anthropometric assessments to the assessors and the statistician during the effect and sensitivity analysis of both the intervention and control group.

### Statistical methods

Baseline characteristics were described using median and interquartile range or counts and percentages for continuous and categorical variables, respectively. A linear mixed model analysis with random intercept unstructured covariance of the repeated measures was applied. The same model based on the binomial distribution with logit link was applied for dichotomous outcomes. Hb values were corrected for altitude^(27)^ while PF and sTfR were corrected for inflammation using the Biomarkers Reflecting Inflammation and Nutritional Determinants of Anaemia regression for preschool-aged children,^(31,32)^ and this was also log-transformed. Dichotomous variables were created for anaemia, ID, IDA and IDE^(27–29)^. Adherence to egg intake by the intervention group was calculated as the ratio between the days in which egg was eaten and the overall treatment days^(33)^. All analyses were carried out according to intention-to-treat using linear mixed-effect models with missing values treated as full maximum likelihood according to the ignorable analysis^(34)^. The analysis of time to achieve the 10th and 11th WHO milestones was performed using a non-parametric time-to-event analysis by deriving hazard functions for participants receiving the intervention and the control. The hazard functions were compared using Gray’s test for Equality of Cumulative Incidence Functions. Hazard ratio was performed using the Cox model.

### Sensitivity analysis

Sensitivity analyses were done by excluding infants with low birthweight (birthweight < 2·5 kg), those with Hb, PF and sTfR out of the range of ± 3 s d, and those with LAZ < –6 or > +6, WAZ < –6 or > +5 and WLZ < –5 or > +6^(35)^. Per protocol analyses were also done using mixed models applied to the set of data, excluding participants with missing values at any of the three time points. All statistical tests were two-tailed and considered a type-I error rate of 5 % (*α* = 0·05). The statistical analyses and data management were performed using SAS vers. 9.04.

## Results

**Fig. 1:**
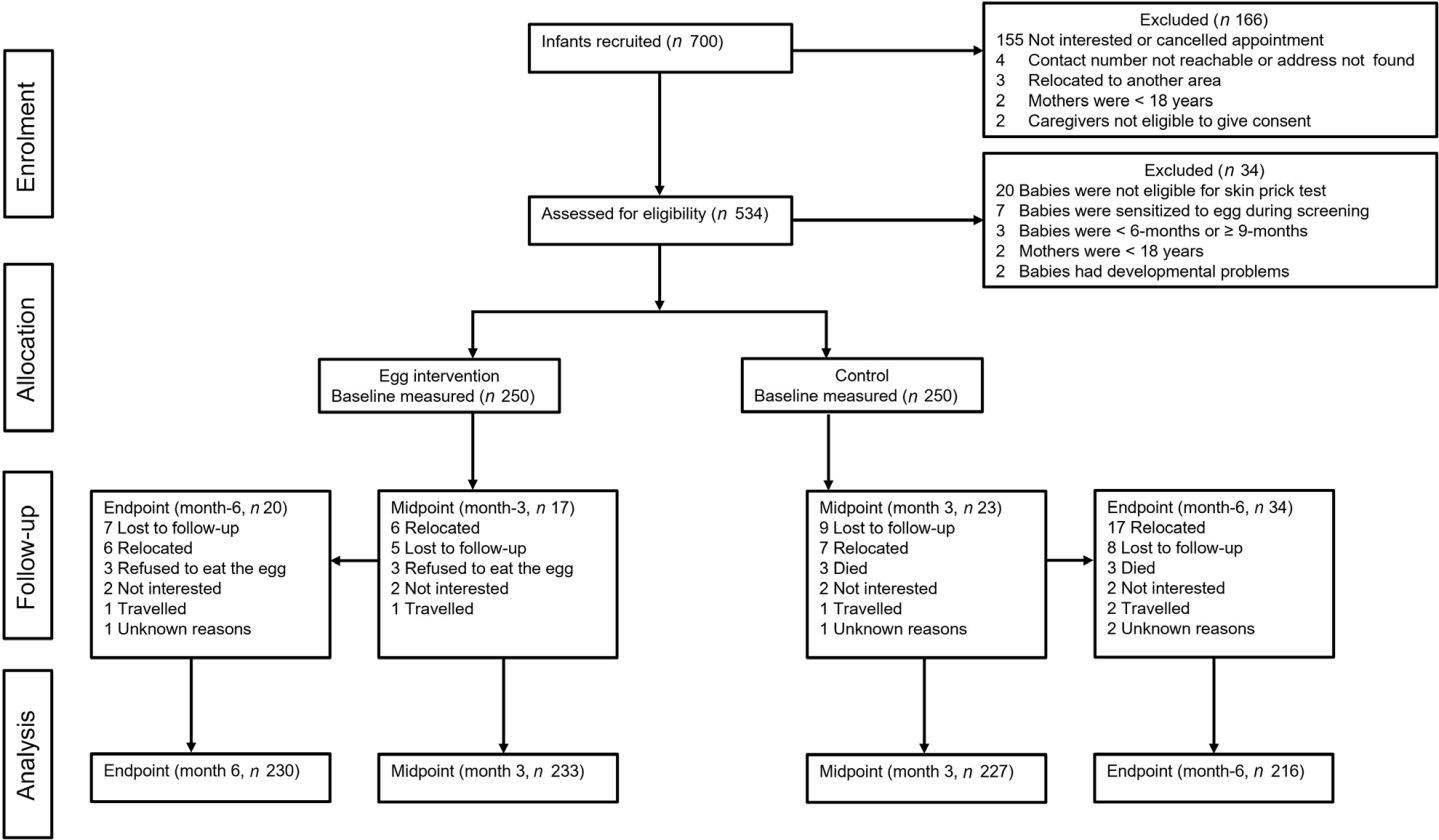
Flow chart of study participants

Figure 1 provides the flow chart of the trial. Recruitment and enrolment of participants took place from February to July 2022. Overall, 700 mother-infant pairs were recruited, of which 534 gave consent to be screened for eligibility. Of these, 500 met the eligibility criteria and were randomly assigned to either the intervention or the control group. Of the 500 mother-infant pairs enrolled, 444 (88·4 %) were the biological mothers of the infants and fifty-eight (11·6 %) were caregivers (thirty-six grandmothers, eighteen aunts, two non-related caregivers, one father, one sibling). Overall, fifty-four (10·8 %) infants dropped out of the study: fifteen were lost to follow-up, twenty-three relocated, three refused to eat the egg, four mothers lost interest in continuing in the study, three died, three travelled, and three were unknown reasons. It is worth mentioning here that due to slower enrolment than anticipated, the six months of follow-up for forty-four mother-infant pairs (intervention group = 27, control group = 17) fell within the upcoming Christmas and New Year holidays. Thus, to avoid a higher dropout rate, their exit measurements were taken two weeks earlier than planned as they may be unreachable after the holidays are over.

### Baseline analysis

Table 1 presents the baseline characteristics of the study participants, with no difference between the intervention and control groups. Although 446 (89·2 %) infants completed the six months of follow-up, the total sample enrolled (*n* 500) was used for the intention-to-treat analysis. Infants had a median (interquartile range) age of 6·54 (6·14, 7·62) months, while that of the mothers was twenty-eight (23, 34) years. Overall, 308 (61·6 %) infants were breastfeeding at baseline, whereas 250 (50·0 %) were receiving infant formula milk every day. Eighty-four (16·8 %) infants presented with low birthweight. Access to own electricity in the households was almost universal (*n* 430; 86·0 %). In the overall group, the prevalence of stunting was 23·8 %, underweight was 9·8 %, wasting was 1·2 %, overweight was 13·8 %, MUACZ < –2 was 0·8 % and HCZ < –2 was 3·4 %. The prevalence of anaemia was 29·2 %, ID was 8·7 %, IDA was 5·4 % and IDE was 43·9 %. Among inflammatory markers assessed, elevated C-reactive protein and *α*-1-acid glycoprotein were observed in fifty-two (11·6 %) and 206 (46·1 %) of the infants, respectively.

**Table 1:** Baseline characteristics of participants by intervention group^a^

	Egg group (*n* 250)		Control group (*n* 250)	
Infant characteristics and anthropometric status	Median	IQR	Median	IQR
Age, months	6·46	6·11, 7·66	6·57	6·14, 7·52
Girls				
* n*	133		125	
%	53·2		50·0	
Breastfeeding rate				
* n*	151		157	
%	60·4		62·8	
Gestational age, weeks	39	38, 40	39	38, 40
Low birthweight (< 2·5 kg)				
* n*	38		46	
%	15·2		18·4	
Weight, kg	7·60	6·79, 8·31	7·63	6·93, 8·42
Length, cm	65·0	63·0, 67·05	65·07	63·30, 67·0
Mid-upper arm circumference, cm	14·85	14·0, 15·65	15·0	13·85, 15·85
Head circumference, cm	43·40	42·30, 44·15	43·23	42·0, 44·40
LAZ	−1·21	–2·01, –0·55	−1·13	–1·87, –0·52
WAZ	−0·31	–1·31, 0·38	−0·29	–1·23, 0·56
WLZ	0·51	–0·15, 1·33	0·59	–0·24, 1·39
MUACZ	0·65	–0·11, 1·32	0·67	–0·28, 1·47
HCZ	0·02	–0·66, 0·66	−0·05	–0·70, 0·61
	*n*	%	*n*	%
Stunting	63	25·2	56	22·4
Underweight	26	10·4	23	9·2
Wasting	4	1·6	2	0·8
Overweight (WLZ > 2)	29	11·6	40	16·0
	Median	IQR	Median	IQR
Iron status				
Hb, g/dl^a^	11·60	10·80, 12·20	11·50	10·80, 12·20
Anaemia (Hb < 11 g/dl)				
* n*	71		75	
%	28·40		30·0	
C-reactive protein	0·47	0·17, 1·87	0·39	0·15, 1·47
*α* 1-glycoprotein	0·94	0·70, 1·50	0·97	0·73, 1·32
Plasma ferritin (PF), μg/l^b^	38·75	21·84, 62·95	38·04	22·28, 66·44
Soluble transferrin receptor (sTfR), mg/l^c^	7·77	6·07, 10·61	8·03	6·22, 11·01
Iron deficiency (ID), (PF < 12 μg/l)				
* n*	20		19	
%	8·6		8·9	
IDA (PF < 12 μg/l and Hb < 11 g/dl)				
* n*	10		11	
%	4·3		5·1	
IDE (sTfR > 8·3 mg/l)				
* n*	107		89	
%	45·9		41·6	
Mother/Caregiver characteristics				
Age, years	29	23, 36	27	23, 33
BMI, kg/m	28·33	23·21, 33·64	27·15	22·86, 33·23
Education (Grade 10 or higher)				
* n*	194		209	
%	77·6		83·6	
Any marriage (Traditional or by law)				
* n*	34		22	
%	13·6		8·8	
Household Characteristics				
Number of people in the household	5	4, 7	5	4, 7
Number of primary school children	1	0, 2	1	0, 2
Number of children below 5 years	1	1, 2	1	1, 2
Number of people employed/receiving^c^ salary	1	0, 1	1	0, 1
Number of people receiving social grants^d^	3	1, 4	2	2, 3
Flush toilet^e^				
* n*	228		224	
%	91·2		89·6	
Own tap water				
* n*	103		113	
%	41·2		45·2	

LAZ: length-for-age Z-score, WAZ: weight-for-age Z-score, WLZ: weight-for-length Z-score, MUACZ: Mid-upper arm circumference-for-age Z-score, HCZ: head circumference-for-age Z-score, IDA: iron deficiency anaemia, IDE: iron deficiency erythropoiesis.

a Corrected for altitude using a factor of –0.2(27).

b Corrected for inflammation using the BRINDA method (31, 32).

c Values presented as median and interquartile range and all such values, unless specified.

d Includes child, old age pension and disability grant.

e Both inside and outside the house.

### Intervention outcomes and estimations

**Fig. 2:**
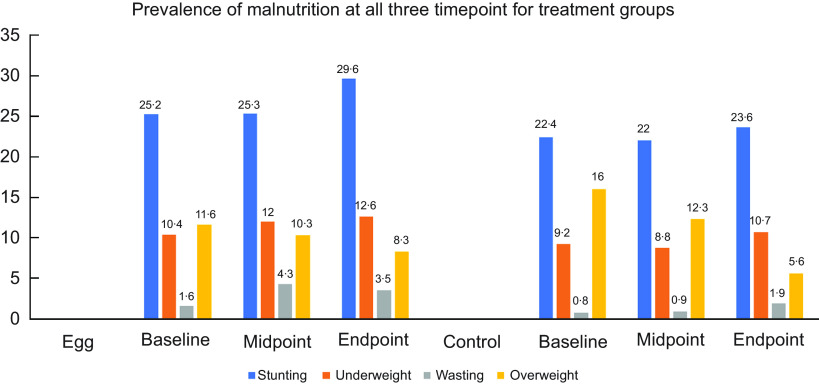
Prevalence of malnutrition at all three time points

Except for the significant increase in egg consumption in the intervention group compared to the control group, intake of other animal-source food did not differ between groups (Table 2). Figure 2 presents the prevalence of stunting, underweight, wasting and overweight at all three time points, with no difference between groups. At the end of the study, the overall prevalence of stunting was 26·7 %, underweight 11·7 %, wasting 2·7 %, overweight declined to 7·0 %, MUACZ < –2 was 1·6 % and HCZ < –2 was 5·4 %. Table 3 presents the primary and secondary outcomes of the study. The primary aim of this study was to examine the effect of egg consumption on LAZ. At the end of the six-month follow-up period, there was no significant effect on LAZ in the egg group compared to the control group (*P* = 0·4648). Likewise, there was no significant effect on the prevalence of stunting (OR = 1·36; 95 % CL: 0·89, 2·08; *P* = 0·1572). Similarly, there was no significant effect on secondary anthropometric outcomes (WAZ, WLZ, MUACZ, HCZ) and time to reach the 10th and 11th WHO gross motor milestones. The overall prevalence of anaemia at the end of the six months of follow-up increased to 35·4 %, ID to 22·7 %, IDA was 13·9 % and IDE to 57·4 %, with no difference between groups. Overall, elevated C-reactive protein and *α*-1-acid glycoprotein were observed in 70 (15·9 %) and 259 (58·7 %) of the infants, respectively, with no difference between groups. The estimated adherence to daily egg intake by the intervention group was 96·3 %.

**Table 2:** Proportion of animal-source food intake by treatment group

		Egg group								Control group								
		*n*	%	*n*	%	*n*	%	*n*	%	*n*	%	*n*	%	*n*	%	*n*	%	
	*n*	7 d/week		4–6 d/week		1–3 d/week		Never		7 d/week		4–6 d/week		1–3 d/week		Never		*P*-value
Infant formula																		
Baseline	500	127	50·8	7	2·8	7	2·8	109	43·6	123	49·2	7	2·8	14	5·6	106	42·4	0·5055
Midpoint	460	109	46·8	5	2·2	2	0·9	117	50·2	93	41	7	3·1	7	3·1	120	52·9	0·2261
Endpoint	446	84	36·5	3	1·3	8	3·5	135	58·7	73	33·8	3	1·4	6	2·8	134	62·0	0·9044
Milk																		
Baseline	500	6	2·4	3	2·4	66	26·4	175	26·4	11	4·4	5	2·0	56	22·4	178	22·4	0·4280
Midpoint	460	33	14·2	13	5·6	101	43·3	86	36·9	33	14·5	11	4·9	102	44·9	81	35·7	0·9714
Endpoint	446	54	23·5	19	8·3	101	43·9	56	24·3	37	17·1	11	5·1	118	54·6	50	23·1	0·0896
Yoghurt																		
Baseline	500	1	0·4	2	0·8	84	33·6	163	65·2	3	1·2	5	2·0	73	29·2	169	67·6	0·3800
Midpoint	460	7	3·0	11	4·7	110	47·2	105	45·1	9	4·0	11	4·9	114	45·8	103	45·4	0·9563
Endpoint	446	10	4·4	20	8·7	118	51·3	82	35·7	12	5·6	21	9·7	105	48·6	78	36·1	0·8876
Chicken																		
Baseline	500	1	0·4	10	4·0	52	20·8	187	74·8	0	0·0	11	4·4	60	24·0	179	71·6	0·6637
Midpoint	460	9	3·9	22	9·4	103	44·2	99	42·5	6	2·6	33	14·5	100	44·1	88	38·8	0·3369
Endpoint	446	10	4·4	36	15·7	112	48·7	72	31·3	10	4·6	40	18·5	102	47·2	64	29·6	0·8723
Meat																		
Baseline	250	0	0·0	0	0·0	13	5·2	237	94·8	0	0·0	0	0·0	11	4·4	239	95·6	0·8348
Midpoint	460	0	0·0	0	0·0	35	15·0	198	85·0	0	0·0	2	0·9	49	21·6	176	77·5	0·0453
Endpoint	446	0	0·0	2	0·9	63	27·4	165	71·7	0	0·0	4	1·9	80	37·0	132	61·1	0·0518
Liver																		
Baseline	500	2	0·8	1	0·4	35	14·0	212	84·8	0	0·0	2	0·8	38	15·2	210	84·0	0·6078
Midpoint	460	2	0·9	0	0·0	62	26·6	169	72·5	0	0·0	2	0·9	71	31·3	154	67·8	0·1295
Endpoint	446	0	0·0	0	0·0	72	31·3	158	68·7	0	0·0	2	0·9	68	31·5	146	67·6	0·4874
Fish																		
Baseline	499	0	0·0	0	0·0	11	4·4	239	95·6	0	0·0	0	0·0	9	3·6	240	96·4	0·8202
Midpoint	460	0	0·0	1	0·4	40	17·2	192	82·4	0	0·0	1	0·4	32	14·1	194	85·5	0·6856
Endpoint	446	0	0·0	0	0·0	64	27·8	166	72·2	0	0·0	0	0·0	60	27·8	156	72·2	1·0000
Egg																		
Baseline	499	3	1·2	9	3·6	108	43·2	130	52·0	5	2·0	8	3·2	81	32·5	155	62·2	0·0786
Midpoint	460	171	73·4	34	14·6	18	7·7	10	4·3	6	2·6	20	8·8	119	52·4	82	36·1	<0·0001*
Endpoint	446	142	61·7	47	20·4	33	14·3	8	3·5	10	4·6	19	8·8	114	52·8	73	33·8	<0·0001*

*P*-value based on the frequency of animal-source food consumption between egg and control group at each time point.

* *P* < 0·01. nr: number.

**Table 3:** Effect of egg intervention on anthropometric, Hb and iron status according to intention-to-treat analysis†

Outcomes at midpoint	Egg group (*n* 233)		Control group (*n* 227)		Effect**	95 % CI	*P*-value
	Median	IQR	Median	IQR	Median	IQR	
Length, cm	69·31	68·91, 69·70	69·30	68·90, 69·70	0·01	–0·56, 0·57	0·9822
LAZ	−1·23	–1·38, –1·08	−1·23	–1·38, –1·08	−0·01	–0·22, 0·21	0·9571
Stunting							
* n*	59		50		1·20	0·78, 1·85	0·4074
%	25·3		22·0				
Weight, kg	8·51	8·34, 8·69	8·61	8·43, 8·79	−0·10	–0·34, 0·15	0·4547
WAZ	−0·38	–0·55, –0·21	−0·28	–0·45, –0·10	−0·11	–0·35, 0·14	0·3844
Underweight							
* n*	28		20		1·41	0·77, 2·59	0·2636
%	12·0		8·8				
WLZ	0·40	0·23, 0·56	0·53	0·37, 0·70	−0·14	–0·37, 0·10	0·2506
Wasting							
* n*	10		2		5·04	1·09, 23·34	0·0385
%	4·3		0·9				
Mid-upper arm circumference, cm	15·13	14·95, 15·30	15·17	14·99, 15·35	−0·04	–0·29, 0·21	0·7542
MUACZ	0·62	0·47, 0·77	0·66	0·51, 0·80	−0·04	–0·25, 0·17	0·7305
Head circumference, cm	44·90	44·71, 45·09	44·84	44·65, 45·03	0·06	–0·21, 0·33	0·6749
HCZ	0·12	–0·01, 0·26	0·07	–0·07, 0·20	0·05	–0·14, 0·24	0·5738
Outcomes at endpoint	Egg group (*n* 230)		Control group (*n* 216)		Effect	95 % CI	*P*-value
Length, cm	72·33	71·93, 72·73	72·47	72·06, 72·89	−0·14	–0·72, 0·43	0·6225
LAZ	−1·41	–1·56, –1·26	−1·33	–1·49, –1·18	−0·08	–0·30, 0·13	0·4648
Stunting							
* n*	68		51		1·36	0·89, 2·08	0·1572
%	29·6		23·6				
Weight, kg	9·05	8·88, 9·23	9·14	8·96, 9·32	−0·08	–0·34, 0·17	0·5157
WAZ	−0·51	–0·69, –0·34	−0·41	–0·59, –0·24	−0·10	–0·35, 0·15	0·4250
Underweight							
* n*	29		23		1·21	0·68, 2·17	0·5206
%	12·6		10·7				
WLZ	0·23	0·06, 0·39	0·31	0·14, 0·48	−0·08	–0·32, 0·16	0·5102
Wasting							
* n*	8		4		1·91	0·57, 6·45	0·2976
%	3·5		1·9				
Mid-upper arm circumference, cm	15·30	15·12, 15·48	15·30	15·11, 15·49	0·00	–0·26, 0·26	0·9987
MUACZ	0·66	0·51, 0·81	0·66	0·51, 0·81	0·00	–0·21, 0·21	0·9856
Head circumference, cm	45·32	45·12, 45·51	45·23	45·03, 45·43	0·09	–0·19, 0·36	0·5322
HCZ	−0·28	–0·42, –0·15	−0·35	–0·49, –0·21	0·06	–0·13, 0·26	0·5166
Gross motor milestone development							
Standing without support							
* n*	179		179		0·95	0·77, 1·17	0·6352
%	71·6		71·6				
Walking without support							
* n*	116		113		1·01	0·78, 1·30	0·9655
%	46·4		45·2				
Hb and iron status							
Hb, g/dl‡	11·29	11·15, 11·43	11·34	11·19, 11·49	−0·05	–0·25, 0·16	0·6474
Anaemia (Hb < 11 g/dl)							
* n*	88		70		1·29	0·87, 1·91	0·1982
%	38·3		32·4				
Plasma ferritin (PF), μg/l*,§	22·71	20·08, 25·69	22·58	19·86, 25·66	−0·13	–3·17, 5·17	0·9498
ID (PF < 12 μg/l)							
* n*	51		49		0·95	0·61, 1·49	0·8333
%	22·3		23·1				
IDA (PF < 12 μg/l and Hb 11 g/dl)							
* n*	35		26		1·28	0·74, 2·22	0·3715
%	15·3		12·3				
Soluble transferrin receptor (sTfR), mg/l*,§	9·73	9·11, 10·38	10·21	9·54, 10·93	−0·49	–1·27, 0·51	0·3104
IDE (sTfR > 8·3 mg/l)							
* n*	127		126		0·85	0·58, 1·24	0·4004
%	55·5		59·4				

LAZ: length-for-age Z-score, WAZ: weight-for-age Z-score, WLZ: weight-for-length Z-score, MUACZ: Mid-upper arm circumference-for-age Z-score, HCZ: head circumference-for-age Z-score, IDA: iron deficiency anaemia, IDE: iron deficiency erythropoiesis.

* Geometric means, with analysis performed on log-transformed data.

† Values presented as median and interquartile range and all such values, unless specified.

‡ Corrected for altitude using a factor of –0·2^(27)^.

§ Corrected for inflammation using the Biomarkers Reflecting Inflammation and Nutritional Determinants of Anaemia method^(31,32)^.

** Effects reported as OR for stunting, underweight, wasting and overweight. Effects reported as HR for gross motor milestone development.

### Ancillary analyses

No statistically significant difference was observed for primary and secondary outcomes of interests when sensitivity analyses were done by excluding infants with low birthweight (Supplementary Table 1), anthropometric Z-scores, Hb and iron status indicators out of the range of ± 3 sd (online Supplementary Table 2), as well as by per protocol analysis (online Supplementary Table 3). Furthermore, no effect was observed by excluding infants with LAZ < –6 or > 6, WAZ < –6 or > 5 and WLZ < –5 or +5 (results not shown). The proportion of allergic sensitisation to egg was similar in both the intervention (1·9 %) and control group (1·5 %) at the endpoint (*P*-value = 0·929). A supplementary analysis was done considering primary outcomes in relation to season using a season for treatment interaction term. This analysis showed no statistically significant seasonal effect for the outcomes and for the treatments (data not shown).

### Adverse events

There was no difference between the incidence of adverse events among the intervention and control groups. Only eight (1·6 %) infants experienced severe adverse events, defined based on hospitalisation, six (2·4 %) infants in the intervention group and two (0·8 %) in the control group. There were no egg-related serious adverse events reported. Although three (0·6 %) infants died while in the study, the cause of death was not related to the study, and they all occurred in the control group. Nutrient adequacy, morbidity and allergy symptoms will be examined in detail in future analyses.

## Discussion

This randomised controlled trial investigated the effects of daily consumption of an egg for a period of six months on growth, gross motor milestone development, anaemia and iron status of 6- to 9-month-old infants from a low socioeconomic community in South Africa. We found no significant intervention effect on linear growth (LAZ) and stunting prevalence – the primary outcomes of interest. Similarly, there was no effect found on our secondary outcomes of interest, namely WAZ, WLZ, MUACZ, HCZ, gross motor milestones development (standing and walking without support), anaemia or iron status. The findings of this study agree with a similar Malawian study, where it was found that the provision of an egg per day for a period of six months to infants had no overall intervention effect on linear growth and stunting prevalence^(14)^ nor anaemia and iron status^(15)^.

Overall, our estimated prevalence of stunting, underweight and wasting at study completion was similar to that reported in the 2016 South African Demographic and Health Survey^(18)^. According to the 2016 South African Demographic and Health Survey, stunting was higher in infants from age 9 months compared to age 6 to 8 months, while the probability of being underweight increased with age^(18)^. A study by Smuts *et al.* ^(19)^ in the same study area showed that at age 6 months, the prevalence of stunting was 29·5 %, and underweight was 11·3 % at baseline. At study completion, and when the children were 12 months old, the prevalence of stunting was 38·3 % and underweight was 12·5 %. Although the changes from baseline to follow-up were small, we can reason that the probability of being stunted and/or underweight increase as children grow, especially in the first one thousand days of life^(18,19)^. Thus, complementary feeding interventions are needed in the first two years of life to prevent undernutrition with a specific focus on stunting and underweight reduction^(36)^.

An egg intervention study by lannotti *et al.* ^(10)^, in five rural parishes in a province in Ecuador, found that the provision of an egg per day increased LAZ, WAZ and reduced the prevalence of stunting and underweight by 47 % and 74 %, respectively, which disagrees with our findings. Additionally, a systematic review and meta-analysis by Asare *et al.* ^(37)^ found that animal-source food supplementation increased LAZ and WAZ, especially when the animal-source food was egg, which also disagrees with our findings. The lack of intervention effect in the current study may be because the addition of one egg to the usual complementary diet with low dietary diversity of the infants was insufficient to meet the demands for improved and sustained growth during the six-month intervention period^(16)^. Nevertheless, this does not exclude the fact that eggs are affordable^(38)^ and widely accessible complementary food. It has been shown that providing one egg a day for six months did not increase adverse events, nor lead to more allergic sensitisation in the egg group, nor displaced other complementary foods^(16,39)^. Therefore, it is still one of the many animal-source foods rich in macro- and micronutrients needed for optimal child growth and development, especially in food-insecure settings^(7–10)^.

There was no intervention effect on the infants’ ability to stand and walk without support. Our finding is similar to that of Prado *et al.*, who failed to show egg intervention effect on child’s fine and gross motor, language development, and personal and social development^(40)^. On the other hand, it could be reasoned that the crudeness of the measures adopted to assess motor development may have influenced our outcome. These measures did not assess other developmental outcomes, such as fine motor, language, personal and social development. Measures to assess these developmental outcomes may have provided different results. This notwithstanding, both growth (irreversible constant increase in body size) and development (growth in psychomotor capacity) associate with each other and are dependent on nutrition, genetic and environmental factors^(41)^. Thus, we can speculate that the provision of one egg per day in addition to the normal dietary intake of the infants together with the duration of the intervention does not make that much of a difference to affect gross motor milestones development.

Although there was no intervention effect on anaemia or iron status (PF and sTfR), there was an increase in the overall prevalence of anaemia, ID, IDA and IDE at the end of the six months of follow-up period, and the prevalence rates signify a moderate to high public health problem^(42)^. Many factors could have had an influence on anaemia or iron status outcomes of this study. Firstly, iron requirements for infants and young children increase with age^(43)^. This subsequently increases the demands for proteins, as it is the main nutrient involved in the overall metabolism of iron^(44)^. Therefore, sufficient intake of both iron and protein is necessary to maintain a normal iron status in the human body^(45)^. One whole cooked egg, irrespective of the cooking method, contains 1·69 mg of iron per 100 g (approximately 0·9 mg per 50 g egg)^(46)^, which is much lower than the RDA for children aged seven to 12 months (3 mg) and 12 to 36 months (7 mg)^(43)^. Thus, we hypothesise that the provision of one egg per day was not sufficient to provide adequate iron to address anaemia or ID in this low socioeconomic community.

Secondly, the whole egg consists of the yolk and egg white or albumen^(7)^, with most of the haem iron in the egg being found in the yolk and some traces in the egg white^(15)^. However, the bioavailability of iron from egg yolk has been shown to be poor, which was in congruence with an animal study by Kobayashi *et al.* ^(45)^, who showed that egg yolk intake did not have effect on IDA because it delayed improvement of liver iron content, transferrin saturation level, Hb and haematocrit. This also agrees with a previous study by Makrides *et al.* ^(13)^, who showed that egg yolk intake did not have any significant intervention effect on Hb, PF and transferrin in breastfed infants aged 6 to 12 months. However, egg yolk intake resulted in the enhancement of plasma iron and transferrin saturation levels. Both egg yolk and egg white have also been shown to influence iron absorption and bioavailability. Iron-containing food, such as beef, has also been shown to increase iron absorption^(47,48)^. Therefore, it is possible that the absorption of dietary iron, in particular non-haem iron, may have been inhibited by other dietary components in egg as shown by previous studies^(45,48,49)^, which may explain the lack of intervention effect on anaemia or iron status in our study.

Smuts *et al.* ^(19)^ supplemented 6- to 12-month-old infants with two different small-quantity lipid-based nutrient supplements (SQ-LNS) products: SQ-LNS and SQ-LNS-plus with an iron contribution of 5·8 mg each. This is much higher than iron provided by egg and could have influenced their outcomes, as intervention with both supplement products increased Hb concentration and reduced the risk of anaemia, ID and IDA in the North West province of South Africa. The SQ-LNS also contain high amounts of energy, vitamins, minerals and essential fatty acids, such as vitamin C, zinc and *α*-linolenic acid^(19)^. Therefore, it is possible that the high contribution of vitamin C from the SQ-LNS (SQ-LNS = 23·3 mg; SQ-LNS-plus = 103 mg) and phytase from SQ-LNS-plus (200 FTU, phytase activity unit) might have enhanced iron absorption, especially by releasing non-haem iron bound to anti-nutrients in complementary foods to influence anaemia and iron status biomarkers^(43)^. As egg does not contain vitamin C^(7)^, we can hypothesise that the absorption of non-haem iron was inhibited, especially because most of the usual complementary diets of infants and young children contain inhibitors of non-haem iron absorption, such as phytate and tannins^(43)^. Lastly, although the randomisation generated similar groups, we speculate that chronic lack of micronutrients or hidden hunger may have influenced the outcome of this study. Except for iron status assessment, this study did not assess other micronutrients in detail and cannot ascertain if hidden hunger played a role in the outcomes of our study; therefore, it should be investigated in future studies of this nature^(50)^.

This study has some limitations. Firstly, there was no direct observation of egg intake by infants in the intervention group but reported by the mother in an adherence diary. Thus, we cannot exclude the fact that there may have been sharing of the weekly ration of eggs with other household members, which could have attenuated our outcomes. Secondly, although the field workers received training to collect information on gross motor milestones development, the self-reported nature of the date on which a developmental milestone was achieved by the mothers may have impacted on the accuracy of the developmental outcome. Again, it could be speculated that assessing gross motor milestones development may require more than six-month intervention period, thus assessing other developmental outcomes, such as fine motor, language, social development could have provided different results. Lastly, it could be that the provision of one egg per day in addition to the normal dietary intake of the infants does not make a big enough difference to impact linear growth and development.

This notwithstanding, this study has many strengths. The study was well-powered to detect small effects due to the large sample size and low rate of dropout. There were weekly household visits to stock up the intervention eggs, check for allergies and morbidity symptoms and to ensure adherence to taking the study eggs. To avoid intra-household sharing of the weekly ration of seven eggs for the infants in the intervention group, there was an additional five eggs provided to each household every week. In addition, this study provided the household of the control group with 5 kg maize meal every month during the six months of follow-up intervention period and 4 dozen eggs at the endpoint, which may have been an incentive for those in the control group to remain in the study. Lastly, although not completely blinded due to the study’s nature, there was full blinding of the field assessors and statistician to the treatment assignment and effect size analysis.

In conclusion, daily egg intake for six months in 6- to 9-month-old infants did not have any overall intervention effect on linear growth (LAZ), stunting prevalence, gross motor milestones development, anaemia, or iron status. Our study showed that relying solely on the intervention of daily egg consumption may not be sufficient to address the growth, development and nutritional status of infants and young children in low socioeconomic communities. This suggests that additional approaches and interventions should be considered to complement or enhance the effects of egg consumption, such as incorporating other dietary sources, improving dietary diversity or implementing strategies to enhance nutrient absorption and bioavailability.

## Supporting information

Ricci et al. supplementary material 1 Ricci et al. supplementary material

Ricci et al. supplementary material 2 Ricci et al. supplementary material

Ricci et al. supplementary material 3 Ricci et al. supplementary material

## Supplementary material

For supplementary material accompanying this paper visit https://doi.org/10.1017/S1368980023002604
