# Supplementary material for: Effects of egg as an early complementary food on growth of 6- to 9-month-old infants: a randomised controlled trial
Source: Public Health Nutr. 2023 Nov 29;27(1):e1. doi: 10.1017/S1368980023002604 (PMC10830362; doi:10.1017/S1368980023002604)
Supplement: Ricci et al. supplementary material 1 — Ricci et al. supplementary material [file S1368980023002604sup001.docx]

Supplementary Table 1. Prevalence and mean difference between treatment groups excluding low birthweight infants^a^

| *Outcomes at midpoint* |  | Egg group (n = 197) |  | Control group (n = 185) |  | **Effect (95% Cl) |  | P-value |
| --- | --- | --- | --- | --- | --- | --- | --- | --- |
| Length, cm |  | 69.81 (69.41, 70.22) |  | 69.66 (69.24, 70.08) |  | 0.15 (-0.43, 0.74) |  | 0.6041 |
| LAZ |  | -1.01 (-1.16, -0.87) |  | -1.09 (-1.24, -0.94) |  | 0.08 (-0.13, 0.29) |  | 0.4691 |
| Stunting, n (%) |  | 39 (19.8) |  | 31 (16.8) |  | 1.28 (0.61, 2.70) |  | 0.5115 |
| Weight, kg |  | 8.64 (8.45, 8.83) |  | 8.71 (8.52, 8.90) |  | -0.07 (-0.34, 0.19) |  | 0.5879 |
| WAZ |  | -0.24 (-0.41, -0.06) |  | -0.18 (-0.35, 0.00) |  | -0.06 (-0.31, 0.19) |  | 0.6311 |
| Underweight, n (%) |  | 17 (8.6) |  | 12 (6.5) |  | 1.44 (0.53, 3.91) |  | 0.4761 |
| WLZ |  | 0.43 (0.26, 0.60) |  | 0.56 (0.39, 0.73) |  | -0.13 (-0.37, 0.11) |  | 0.3004 |
| Wasting, n (%) |  | 7 (3.6) |  | 1 (0.50) |  | Not estimable |  | - |
| Mid-upper arm circumference, cm |  | 15.19 (15.00, 15.37) |  | 15.22 (15.03, 15.41) |  | -0.04 (-0.30, 0.23) |  | 0.7836 |
| MUACZ |  | 0.68 (0.53, 0.83) |  | 0.70 (0.55, 0.85) |  | -0.02 (-0.23, 0.20) |  | 0.8687 |
| Head circumference, cm |  | 45.05 (44.86, 45.24) |  | 45.00 (44.80, 45.19) |  | 0.05 (-0.22, 0.32) |  | 0.6941 |
| HCZ |  | 0.25 (0.12, 0.38) |  | 0.18 (0.05, 0.31) |  | 0.07 (-0.11, 0.25) |  | 0.4406 |
| *Outcomes at endpoint* |  | Egg (n = 194) |  | Control (n = 175) |  | Effect (95% Cl) |  | P-value |
| Length, cm |  | 72.86 (72.43, 73.29) |  | 72.76 (72.32, 73.20) |  | 0.10 (-0.52, 0.72) |  | 0.7521 |
| LAZ |  | -1.19 (-1.34, -1.04) |  | -1.25 (-1.41, -1.10) |  | 0.06 (-0.16, 0.28) |  | 0.5848 |
| Stunting, n (%) |  | 47 (24.2) |  | 33 (18.9) |  | 1.45 (0.70, 2.98) |  | 0.3139 |
| Weight, kg |  | 9.19 (8.99, 9.39) |  | 9.19 (8.98, 9.39) |  | 0.00 (-0.28, 0.29) |  | 0.9730 |
| WAZ |  | -0.36 (-0.53, -0.19) |  | -0.38 (-0.56, -0.20) |  | 0.02 (-0.23, 0.27) |  | 0.8811 |
| Underweight, n (%) |  | 17 (8.8) |  | 14 (8.0) |  | 1.10 (0.42, 2.88) |  | 0.8511 |
| WLZ |  | 0.29 (0.12, 0.45) |  | 0.30 (0.13, 0.47) |  | -0.01 (-0.25, 0.23) |  | 0.9235 |
| Wasting, n (%) |  | 4 (2.1) |  | 2 (1.1) |  | Not estimable |  | - |
| Mid-upper arm circumference, cm |  | 15.37 (15.18, 15.57) |  | 15.31 (15.11, 15.51) |  | 0.07 (-0.21, 0.34) |  | 0.6384 |
| MUACZ |  | 0.74 (0.59, 0.89) |  | 0.67 (0.51, 0.82) |  | 0.07 (-0.15, 0.29) |  | 0.5192 |
| Head circumference, cm |  | 45.47 (45.28, 45.66) |  | 45.38 (45.19, 45.57) |  | 0.09 (-0.17, 0.36) |  | 0.4934 |
| HCZ |  | -0.15 (-0.28, -0.02) |  | -0.25 (-0.38, -0.12) |  | 0.11 (-0.08, 0.29) |  | 0.2536 |
| ***Gross motor milestone development*** |  |  |  |  |  |  |  |  |
| Standing without support, n (%) |  | 153 (72.2) |  | 149 (73.0) |  | 0.97 (0.71, 1.32) |  | 0.8425 |
| Walking without support, n (%) |  | 104 (49.1) |  | 97 (47.6) |  | 1.03 (0.86, 1.24) |  | 0.7583 |
| ***Haemoglobin (Hb) and iron status*** |  |  |  |  |  |  |  |  |
| Hb, g/dL^b^ |  | 11.32 (11.17, 11.47) |  | 11.34 (11.18, 11.49) |  | -0.01 (-0.23, 0.21) |  | 0.9051 |
| Anaemia (Hb < 11 g/Dl), n (%) |  | 76 (36.2) |  | 55 (31.4) |  | 1.39 (0.87, 2.23) |  | 0.1703 |
| *Plasma ferritin (PF), μg/L^c^ |  | 23.27 (20.31, 26.66) |  | 24.14 (20.90, 27.88) |  | -0.87 (-4.37, 4.89) |  | 0.7175 |
| ID (PF < 12 μg/L), n (%) |  | 42 (21.8) |  | 36 (20.9) |  | 1.05 (0.60, 1.84) |  | 0.8644 |
| IDA (PF < 12 μg/L and Hb < 11 g/dL), n (%) |  | 29 (15.0) |  | 19 (11.1) |  | 1.43 (0.72, 2.85) |  | 0.3094 |
| *Soluble transferrin receptor (sTfR), mg/L^c^ |  | 9.33 (8.68, 10.04) |  | 9.87 (9.14, 10.67) |  | -0.54 (-1.37, 0.54) |  | 0.2981 |
| IDE (sTfR > 8.3 mg/L), n (%) |  | 103 (53.4) |  | 98 (57.0) |  | 0.86 (0.54, 1.38) |  | 0.5325 |
| LAZ: length-for-age Z-score, WAZ: weight-for-age Z-score, WLZ: weight-for-length Z-score, HCZ: head circumference-for-age Z-score, MUACZ: Mid-upper arm-circumference-for-age Z-score, IDA: iron deficiency anaemia, IDE: iron deficiency erythropoiesis.  ^a^Values presented as median and interquartile range and all such values, unless specified.  ^b^Corrected for altitude using a factor of -0.2^(27)^  ^c^Corrected for inflammation using the BRINDA method^(31, 32)^.  *Geometric means, with analysis performed on log transformed data.  **Effects reported as ORs for stunting, underweight, wasting, and overweight.  **Effects reported as HRs for Gross motor milestone development. | | | | | | | | |
