# Supplementary material for: Effects of egg as an early complementary food on growth of 6- to 9-month-old infants: a randomised controlled trial
Source: Public Health Nutr. 2023 Nov 29;27(1):e1. doi: 10.1017/S1368980023002604 (PMC10830362; doi:10.1017/S1368980023002604)
Supplement: Ricci et al. supplementary material 2 — Ricci et al. supplementary material [file S1368980023002604sup002.docx]

Supplementary Table 2. Prevalence and mean difference between treatment groups by excluding extreme values^a^

| *Outcomes at midpoint* |  | Egg group (n = 231) |  | Control group (n = 226) |  | **Effect (95% Cl) |  | P-value |
| --- | --- | --- | --- | --- | --- | --- | --- | --- |
| Length, cm^†^ |  | 69.31 (68.92, 68.92) |  | 69.21 (68.82, 69.61) |  | 0.09 (-0.46, 0.64) |  | 0.7412 |
| LAZ^‡^ |  | -1.05 (-1.18, -0.92) |  | -1.08 (-1.21, -0.95) |  | 0.03 (-0.15, 0.22) |  | 0.7435 |
| Stunting, n (%)^‡^ |  | 42 (19.4) |  | 37 (17.3) |  | 1.15 (0.71, 1.89) |  | 0.5652 |
| Weight, kg† |  | 8.47 (8.30, 8.63) |  | 8.59 (8.42, 8.75) |  | -0.12 (-0.36, 0.12) |  | 0.3171 |
| WAZ^‡^ |  | -0.32 (-0.47, -0.16) |  | -0.25 (-0.41, -0.10) |  | -0.06 (-0.28, 0.16) |  | 0.5730 |
| Underweight, n (%)^‡^ |  | 21 (9.4) |  | 16 (7.2) |  | 1.33 (0.67, 2.62) |  | 0.4170 |
| WLZ^‡^ |  | 0.32 (0.17, 0.46) |  | 0.41 (0.27, 0.56) |  | -0.10 (-0.30, 0.11) |  | 0.3665 |
| Wasting, n (%)^‡^ |  | 10 (4.4) |  | 2 (0.9) |  | 4.98 (1.07, 23.0) |  | 0.0402 |
| Overweight, n (%)^‡^ |  | 19 (15.0) |  | 20 (9.1) |  | 0.90 (0.47, 1.75) |  | 0.7653 |
| Mid-upper arm circumference, cm^†^ |  | 15.09 (14.91, 15.26) |  | 15.15 (14.97, 15.32) |  | -0.06 (-0.31, 0.18) |  | 0.6185 |
| MUACZ^‡^ |  | 0.55 (0.42, 0.69) |  | 0.60 (0.46, 0.74) |  | -0.05 (-0.25, 0.14) |  | 0.6142 |
| Head circumference, cm^†^ |  | 44.90 (44.71, 45.09) |  | 44.82 (44.63, 45.01) |  | 0.08 (-0.18, 0.34) |  | 0.5553 |
| HCZ^‡^ |  | 0.12 (0.00, 0.25) |  | 0.07 (-0.06, 0.20) |  | 0.05 (-0.13, 0.23) |  | 0.5591 |
| *Outcomes at endpoint* |  | Egg (n = 229) |  | Control (n = 214) |  | Effect (95% Cl) |  | P-value |
| Length, cm^†^ |  | 72.33 (71.94, 71.94) |  | 72.42 (72.02, 72.82) |  | -0.09 (-0.65, 0.47) |  | 0.7531 |
| LAZ^‡^ |  | -1.20 (-1.34, -1.07) |  | -1.17 (-1.31, -1.04) |  | -0.03 (-0.22, 0.16) |  | 0.7428 |
| Stunting, n (%)^‡^ |  | 48 (22.9) |  | 36 (17.9) |  | 1.36 (0.84, 2.21) |  | 0.2160 |
| Weight, kg^†^ |  | 9.00 (8.84, 9.17) |  | 9.07 (8.90, 9.24) |  | -0.07 (-0.31, 0.17) |  | 0.5755 |
| WAZ^‡^ |  | -0.42 (-0.58, -0.26) |  | -0.39 (-0.55, -0.23) |  | -0.03 (-0.25, 0.20) |  | 0.8044 |
| Underweight, n (%)^‡^ |  | 20 (9.1) |  | 18 (8.7) |  | 1.06 (0.54, 2.07) |  | 0.8625 |
| WLZ^‡^ |  | 0.19 (0.04, 0.34) |  | 0.20 (0.04, 0.35) |  | -0.01 (-0.22, 0.20) |  | 0.9555 |
| Wasting, n (%)^‡^ |  | 6 (2.7) |  | 4 (1.9) |  | 1.41 (0.39, 5.09) |  | 0.5990 |
| Overweight n (%)^‡^ |  | 8.33 (6.7) |  | 5 (2.4) |  | 2.93 (1.04, 8.22) |  | 0.0414 |
| Mid-upper arm circumference, cm^†^ |  | 15.27 (15.10, 15.45) |  | 15.25 (15.07, 15.43) |  | 0.02 (-0.23, 0.27) |  | 0.8731 |
| MUACZ^‡^ |  | 0.60 (0.46, 0.74) |  | 0.57 (0.43, 0.72) |  | 0.03 (-0.17, 0.23) |  | 0.7992 |
| Head circumference, cm^†^ |  | 45.32 (45.13, 45.50) |  | 45.21 (45.01, 45.40) |  | 0.11 (-0.16, 0.38) |  | 0.4273 |
| HCZ^‡^ |  | -0.26 (-0.39, -0.13) |  | -0.30 (-0.44, -0.17) |  | 0.05 (-0.14, 0.23) |  | 0.6258 |
| ***Haemoglobin (Hb) and iron status*** |  |  |  |  |  |  |  |  |
| Hb, g/dL^b†^ |  | 11.33 (11.19, 11.47) |  | 11.34 (11.19, 11.48) |  | -0.01 (-0.21, 0.19) |  | 0.9026 |
| Anaemia (Hb < 11 g/dL), n (%)^†^ |  | 86 (37.7) |  | 70 (32.4) |  | 1.26 (0.85, 1.87) |  | 0.2429 |
| *Plasma ferritin (PF), μg/L^c†^ |  | 23.85 (21.21, 26.81) |  | 22.58 (20.01, 25.48) |  | 1.27 (-2.15, 6.36) |  | 0.5254 |
| ID (PF < 12 μg/L), n (%)† |  | 48 (21.2) |  | 49 (23.1) |  | 0.90 (0.57, 1.41) |  | 0.6380 |
| IDA (PF < 12 μg/L and Hb < 11 g/dL), n (%)^†^ |  | 30 (13.4) |  | 26 (12.3) |  | 1.10 (0.63, 1.94) |  | 0.7398 |
| *Soluble transferrin receptor (sTfR), mg/L^c†^ |  | 9.57 (9.01, 10.17) |  | 10.13 (9.52, 10.79) |  | -0.56 (-1.27, 0.33) |  | 0.2013 |
| IDE (sTfR > 8.3 mg/L), n (%)^†^ |  | 124 (55.1) |  | 125 (59.2) |  | 0.84 (0.58, 1.24) |  | 0.3853 |
| LAZ: length-for-age Z-score, WAZ: weight-for-age Z-score, WLZ: weight-for-length Z-score, HCZ: head circumference-for-age Z-score, MUACZ: Mid-upper arm-circumference-for-age Z-score, IDA: iron deficiency anaemia, IDE: iron deficiency erythropoiesis.  ^a^Values presented as median and interquartile range and all such values, unless specified.  ^b^Corrected for altitude using a factor of -0.2^(27)^.  ^c^Corrected for inflammation using the BRINDA method^(31, 32)^.  *Geometric means, analysis performed on Log transformed data.  †Excluding infants with values outside 3 standard deviation (std) range.  ‡Excluding infants with Z-scores outside ± 3 std range.  **Effects reported as ORs for stunting, underweight, wasting, and overweight. | | | | | | | | |
