# Supplementary material for: Effects of egg as an early complementary food on growth of 6- to 9-month-old infants: a randomised controlled trial
Source: Public Health Nutr. 2023 Nov 29;27(1):e1. doi: 10.1017/S1368980023002604 (PMC10830362; doi:10.1017/S1368980023002604)
Supplement: Ricci et al. supplementary material 3 — Ricci et al. supplementary material [file S1368980023002604sup003.docx]

Supplementary Table 3. Effect of egg intervention on anthropometric, haemoglobin and iron status according to per protocol analysis^a^

|  |  | Egg group |  | Control group |  | **Effect (95% Cl) |  | P-value |
| --- | --- | --- | --- | --- | --- | --- | --- | --- |
| *Outcomes at midpoint* |  |  |  |  |  |  |  |  |
| Length, cm |  | 69.28 (68.89, 69.67) |  | 69.29 (68.89, 69.70) |  | -0.01 (-0.58, 0.55) |  | 0.9640 |
| LAZ |  | -1.25 (-1.39, -1.10) |  | -1.21 (-1.37, -1.06) |  | -0.03 (-0.25, 0.18) |  | 0.7688 |
| Stunting, n (%) |  | 58 (25.3) |  | 46 (21.5) |  | 1.33 (0.66, 2.67) |  | 0.4196 |
| Weight, kg |  | 8.51 (8.33, 8.69) |  | 8.64 (8.45, 8.83) |  | -0.13 (-0.39, 0.13) |  | 0.3364 |
| WAZ |  | -0.39 (-0.56, -0.21) |  | -0.24 (-0.42, -0.05) |  | -0.15 (-0.40, 0.10) |  | 0.2457 |
| Underweight, n (%) |  | 27 (11.8) |  | 19 (8.9) |  | 1.49 (0.63, 3.54) |  | 0.3649 |
| WLZ |  | 0.40 (0.24, 0.57) |  | 0.58 (0.40, 0.75) |  | -0.17 (-0.41, 0.06) |  | 0.1527 |
| Wasting n (%) |  | 10 (4.4) |  | 2 (0.9) |  | 4.73 (0.87, 25.80) |  | 0.0728 |
| Mid-upper arm circumference, cm |  | 15.12 (14.94, 15.30) |  | 15.20 (15.01, 15.39) |  | -0.07 (-0.33, 0.18) |  | 0.5740 |
| MUACZ |  | 0.62 (0.47, 0.76) |  | 0.68 (0.53, 0.84) |  | -0.07 (-0.28, 0.15) |  | 0.5384 |
| Head circumference, cm |  | 44.89 (44.70, 45.07) |  | 44.84 (44.65, 45.03) |  | 0.05 (-0.22, 0.31) |  | 0.7347 |
| HCZ |  | 0.11 (-0.02, 0.24) |  | 0.08 (-0.05, 0.21) |  | 0.03 (-0.15, 0.22) |  | 0.7326 |
| *Outcomes at endpoint* |  |  |  |  |  |  |  |  |
| Length, cm |  | 72.32 (71.91, 72.73) |  | 72.46 (72.04, 72.89) |  | -0.14 (-0.73, 0.45) |  | 0.6334 |
| LAZ |  | -1.41 (-1.56, -1.27) |  | -1.34 (-1.49, -1.18) |  | -0.08 (-0.29, 0.14) |  | 0.4830 |
| Stunting, n (%) |  | 68 (29.7) |  | 51 (23.8) |  | 1.53 (0.78, 3.00) |  | 0.2133 |
| Weight, kg |  | 9.05 (8.86, 9.24) |  | 9.13 (8.94, 9.33) |  | -0.08 (-0.35, 0.19) |  | 0.5620 |
| WAZ |  | -0.51 (-0.68, -0.34) |  | -0.42 (-0.60, -0.24) |  | -0.10 (-0.34, 0.15) |  | 0.4521 |
| Underweight, n (%) |  | 29 (12.7) |  | 23 (10.8) |  | 1.23 (0.54, 2.79) |  | 0.6214 |
| WLZ |  | 0.23 (0.07, 0.39) |  | 0.30 (0.14, 0.47) |  | -0.07 (-0.31, 0.16) |  | 0.5296 |
| Wasting n (%) |  | 8 (3.5) |  | 4 (1.9) |  | 1.64 (0.41, 6.63) |  | 0.4857 |
| Mid-upper arm circumference, cm |  | 15.30 (15.12, 15.48) |  | 15.29 (15.10, 15.48) |  | 0.01 (-0.26, 0.27) |  | 0.9643 |
| MUACZ |  | 0.66 (0.51, 0.81) |  | 0.66 (0.50, 0.81) |  | 0.01 (-0.21, 0.22) |  | 0.9433 |
| Head circumference |  | 45.32 (45.13, 45.50) |  | 45.24 (45.05, 45.43) |  | 0.08 (-0.19, 0.34) |  | 0.5757 |
| HCZ |  | -0.28 (-0.41, -0.15) |  | -0.34 (-0.47, -0.20) |  | 0.06 (-0.13, 0.24) |  | 0.5455 |
| ***Haemoglobin (Hb) and iron status*** |  |  |  |  |  |  |  |  |
| Hb, g/dL^b^ |  | 11.29 (11.15, 11.43) |  | 11.34 (11.19, 11.49) |  | -0.05 (-0.25, 0.16) |  | 0.6474 |
| Anaemia (Hb < 11 g/dL), n (%) |  | 88 (38.3) |  | 70 (32.4) |  | 1.29 (0.87, 1.91) |  | 0.1982 |
| *Plasma ferritin (PF), μg/L^c^ |  | 22.71 (20.08, 20.08) |  | 22.58 (19.86, 19.86) |  | 0.13 (-3.14, 4.25) |  | 0.9498 |
| ID (PF < 12 μg/L), n (%) |  | 51 (22.3) |  | 49 (23.1) |  | 0.95 (0.61, 1.49) |  | 0.8333 |
| IDA (PF < 12 μg/L and Hb < 11 g/dL), n (%) |  | 35 (15.3) |  | 26 (12.3) |  | 1.28 (0.74, 2.22) |  | 0.3715 |
| *Soluble transferrin receptor (sTfR), mg/L^c^ |  | 9.73 (9.11, 9.11) |  | 10.21 (9.54, 9.54) |  | -0.49 (-1.27, 0.44) |  | 0.3104 |
| IDE (sTfR > 8.3 mg/L), n (%) |  | 127 (55.5) |  | 126 (59.4) |  | 0.85 (0.58, 1.24) |  | 0.4004 |
| LAZ: length-for-age Z-score, WAZ: weight-for-age Z-score, WLZ: weight-for-length Z-score, HCZ: head circumference-for-age Z-score, MUACZ: Mid-upper arm-circumference-for-age Z-score, IDA: iron deficiency anaemia, IDE: iron deficiency erythropoiesis.  ^a^Values presented as median and interquartile range and all such values, unless specified.  ^b^Corrected for altitude using a factor of -0.2^(27)^.  ^c^Corrected for inflammation using the BRINDA method^(31, 32)^  *Geometric means, with analysis performed on log transformed data.  **Effects reported as ORs for stunting, underweight, wasting, and overweight. | | | | | | | | |
